# 2-Amino­pyridinium diphenyl­phosphinate monohydrate

**DOI:** 10.1107/S1600536809033856

**Published:** 2009-08-29

**Authors:** Mohammad Nazari, Alireza Abbasi, Ali Nemati Kharat, Mohammad Reza Hantehzadeh

**Affiliations:** a School of Chemistry, University College of Science, University of Tehran, Tehran, Iran; b Plasma Physics Research Center, Science & Reseach Campus, Islamic Azad University, Tehran, Iran

## Abstract

In the crystal of the title hydrated salt, C_5_H_7_N_2_^+^·C_12_H_10_O_2_P^−^·H_2_O, the cations, anions and water mol­ecules connected by N—H⋯O and O—H⋯O hydrogen bonds into a layer along the *bc* plane; the phenyl rings protrude into the space between the layers. The dihedral angle between rings of anion is 86.1 (1)°.

## Related literature

For bidentate ligands with both hard (nitrogen) and soft (phosphorous) donors, see: Espinet & Soulantica (1999 ▶); Jeffrey & Rauchfuss (1979 ▶). For the use of diphenyl­phosphinic acid in the extraction of trivalent lanthanide cations and as a flame retardant in the ep­oxy resins used in printed circuit boards, see: Almeida (1974 ▶); von Gentzkow *et al.* (1996 ▶); Huber *et al.* (1998 ▶). 
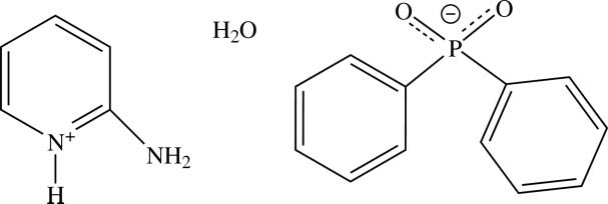


## Experimental

### 

#### Crystal data

C_5_H_7_N_2_^+^·C_12_H_10_O_2_P^−^·H_2_O
*M* *_r_* = 330.31
Monoclinic, 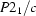

*a* = 15.2716 (19) Å
*b* = 9.979 (2) Å
*c* = 11.7671 (15) Å
β = 103.073 (10)°
*V* = 1746.8 (5) Å^3^
*Z* = 4
Mo *K*α radiation
μ = 0.17 mm^−1^
*T* = 295 K
0.60 × 0.35 × 0.21 mm

#### Data collection

Stoe IPDS-II diffractometer
Absorption correction: analytical (*X-SHAPE*; Stoe & Cie, 2007 ▶) *T*_min_ = 0.813, *T*_max_ = 0.965
8165 measured reflections
2972 independent reflections
1660 reflections with *I* > 2σ(*I*)
*R*_int_ = 0.097

#### Refinement

*R*[*F* ^2^ > 2σ(*F* ^2^)] = 0.054
*wR*(*F* ^2^) = 0.130
*S* = 1.02
2972 reflections
214 parameters
3 restraints
H atoms treated by a mixture of independent and constrained refinement
Δρ_max_ = 0.21 e Å^−3^
Δρ_min_ = −0.20 e Å^−3^

### 

Data collection: *X-RED* (Stoe & Cie, 2007 ▶); cell refinement: *X-AREA* (Stoe & Cie, 2007 ▶); data reduction: *X-AREA*; program(s) used to solve structure: *SHELXS97* (Sheldrick, 2008 ▶); program(s) used to refine structure: *SHELXL97* (Sheldrick, 2008 ▶); molecular graphics: *DIAMOND* (Brandenburg, 2001 ▶); software used to prepare material for publication: *PLATON* (Spek, 2009 ▶).

## Supplementary Material

Crystal structure: contains datablocks I, global. DOI: 10.1107/S1600536809033856/ng2622sup1.cif

Structure factors: contains datablocks I. DOI: 10.1107/S1600536809033856/ng2622Isup2.hkl

Additional supplementary materials:  crystallographic information; 3D view; checkCIF report

## Figures and Tables

**Table 1:** Hydrogen-bond geometry (Å, °)

*D*—H⋯*A*	*D*—H	H⋯*A*	*D*⋯*A*	*D*—H⋯*A*
N1—H1*A*⋯O1	0.86	1.80	2.655 (4)	175
N2—H13⋯O2	0.86	2.02	2.881 (4)	176
N2—H14⋯O3^i^	0.86	2.04	2.853 (4)	157
O3—H3*A*⋯O2^ii^	0.95 (2)	1.79 (2)	2.743 (3)	175 (3)
O3—H3*B*⋯O1	0.95 (2)	1.80 (2)	2.744 (3)	171 (3)

Symmetry codes: (i) 
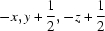
; (ii) 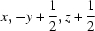.

**Fig. 1.**
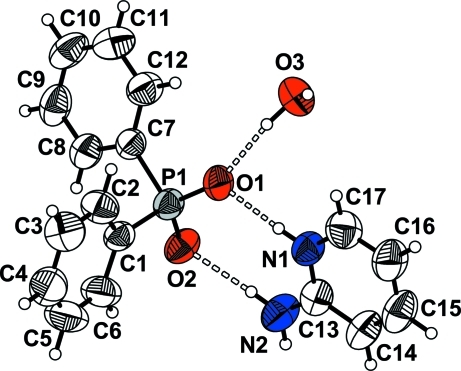
Molecular structure of (I), with 50% probability displacement ellipsoids. H atoms are shown as circles of arbitrary radii.

**Fig. 2.**
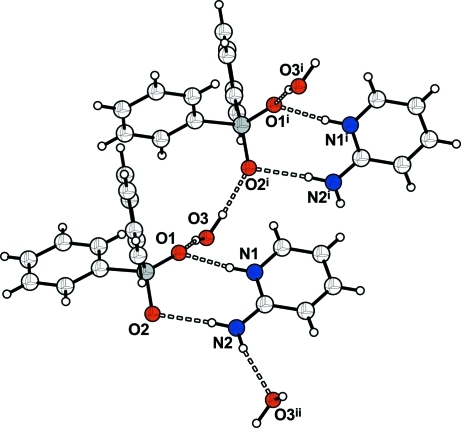
A packing diagram of (I). Hydrogen bonds are shown as dashed lines.

## supplementary crystallographic information

### Comment

Bidentate ligands containing both hard (nitrogen) and soft (phosphorous) donor atoms are extremely fruitful in both homogenous catalysis and coordination chemistry (Espinet & Soulantica, 1999). Because of having both hard and soft donor atoms, they are called hemilabile ligands (Jeffrey & Rauchfuss, 1979). Diphenylphosphinic acid and its derivatives have been widely used because of their variety of applications. It has been extensively used for extraction of trivalent lanthanide cations and as a flame retardant in epoxy resins that are used in printed circuit boards (Almeida, 1974; Huber, *et al.*, 1998; von Gentzkow, *et al.*, 1996).

The molecular structure of (I) and the atom-numbering scheme are shown in Fig. 1. In this work, attempting to get a new hemilabile bidentate ligand, we obtained pyridinium-2-amine di(phenyl)phosphinate monohydrate, which was unexpectedly produced due to the breaking of P—N bond, probably due to its sensitivity to air and humidity. In the crystal structure, there are three discrete moieties in the asymmetric unit (phosphinic acid, pyridine ring and one water molecule) that are in contact by several hydogen bonds, making a well defined motif. There are also C—H···π interactions between phophinate and pyridinium groups between neighboring motifs. Two P—O bonds are slightly different in distances, (P1—O1 = 1.507 Å and P1—O2 = 1.498 Å), that can be due to the hydrogen bonds between the nitrogen atoms of pyridinium rings to the phosphinate molecules (N1—H1A···O1 and N2—H13···O2, 2.655 (4) and 2.881 (4) Å, respectively, see Table 1). The motifs are in contact by hydrogen bonds in *bc* plane, making sheets in which these sheets are held together by van der Waals interactions (see Fig. 2 & 3).

### Experimental

Synthesis was carried out under argon atmosphere at 0°C, by dropwise addition of neat chlorodiphenylphosphine (3.32 g, 15.04 mmol) to a THF solution of 2-aminopyridine (1.41 g, 15.04 mmol) and triethylamine (1.568 g, 15.5 mmol). The mixture was warmed slowly to room temperature, followed by 24 h stirring. Triethylamine hydrochloride precipitates were then filtered off. Removing the excess solvent under reduced pressure, leads to a pale yellow oily product, that was solidifies by solving in benzene and stored in fridge. The obtained solid (0.100 g, 0.359 mmol) together with stoichiometric quantity of sulfur (0.011 g, 0.359 mmol) in toluene were refluxed for 30 minutes and the resulting solution was dried. Recrystallizing in hot toluene afforded colorless needle crystals.

### Refinement

All H atoms (except water molecule) were positioned geometrically [C—H = 0.93Å and N—H = 0.86 (1)Å] and refined using a riding model, with U_iso_(H)=1.2U_eq_(C & N). The H atoms for the water molecules were located from electron density map and refined with a tight restraint of the O-H bond length of 0.95 (2) Å, while keeping the H···H distance at a value corresponding to the H-O-H angle 104^o^.

### Crystal data

**Table:**

C_5_H_7_N_2_^+^·C_12_H_10_O_2_P^−^·H_2_O	*F*(000) = 696
*M**_r_* = 330.31	*D*_x_ = 1.256 Mg m^−^^3^
Monoclinic, *P*2_1_/*c*	Mo *K*α radiation, λ = 0.71073 Å
Hall symbol: -P 2ybc	Cell parameters from 8165 reflections
*a* = 15.2716 (19) Å	θ = 2.5–25.0°
*b* = 9.979 (2) Å	µ = 0.17 mm^−^^1^
*c* = 11.7671 (15) Å	*T* = 295 K
β = 103.073 (10)°	Needle, colorless
*V* = 1746.8 (5) Å^3^	0.60 × 0.35 × 0.21 mm
*Z* = 4	

### Data collection

**Table:**

Stoe IPDS-II diffractometer	2972 independent reflections
Radiation source: fine-focus sealed tube	1660 reflections with *I* > 2σ(*I*)
graphite	*R*_int_ = 0.097
φ oscillation scans	θ_max_ = 25.0°, θ_min_ = 2.5°
Absorption correction: analytical (*X-SHAPE*; Stoe & Cie, 2007)	*h* = −18→17
*T*_min_ = 0.813, *T*_max_ = 0.965	*k* = −11→11
8165 measured reflections	*l* = −13→13

### Refinement

**Table:**

Refinement on *F*^2^	Primary atom site location: structure-invariant direct methods
Least-squares matrix: full	Secondary atom site location: difference Fourier map
*R*[*F*^2^ > 2σ(*F*^2^)] = 0.054	Hydrogen site location: inferred from neighbouring sites
*wR*(*F*^2^) = 0.130	H atoms treated by a mixture of independent and constrained refinement
*S* = 1.01	*w* = 1/[σ^2^(*F*_o_^2^) + (0.05*P*)^2^] where *P* = (*F*_o_^2^ + 2*F*_c_^2^)/3
2972 reflections	(Δ/σ)_max_ < 0.001
214 parameters	Δρ_max_ = 0.21 e Å^−^^3^
3 restraints	Δρ_min_ = −0.20 e Å^−^^3^

### Special details

**Table:**

Geometry. All e.s.d.'s (except the e.s.d. in the dihedral angle between two l.s. planes) are estimated using the full covariance matrix. The cell e.s.d.'s are taken into account individually in the estimation of e.s.d.'s in distances, angles and torsion angles; correlations between e.s.d.'s in cell parameters are only used when they are defined by crystal symmetry. An approximate (isotropic) treatment of cell e.s.d.'s is used for estimating e.s.d.'s involving l.s. planes.
Refinement. Refinement of *F*^2^ against ALL reflections. The weighted *R*-factor *wR* and goodness of fit *S* are based on *F*^2^, conventional *R*-factors *R* are based on *F*, with *F* set to zero for negative *F*^2^. The threshold expression of *F*^2^ > σ(*F*^2^) is used only for calculating *R*-factors(gt) *etc*. and is not relevant to the choice of reflections for refinement. *R*-factors based on *F*^2^ are statistically about twice as large as those based on *F*, and *R*- factors based on ALL data will be even larger.

### Fractional atomic coordinates and isotropic or equivalent isotropic displacement parameters (Å^2^)

**Table:**

	*x*	*y*	*z*	*U*_iso_*/*U*_eq_	
P1	0.23381 (6)	0.38907 (9)	0.28738 (7)	0.0566 (3)	
O1	0.19470 (14)	0.3089 (2)	0.37261 (18)	0.0684 (6)	
O2	0.16905 (13)	0.4311 (2)	0.17762 (16)	0.0679 (6)	
C1	0.2857 (2)	0.5359 (3)	0.3623 (3)	0.0549 (8)	
C2	0.3479 (2)	0.5264 (4)	0.4676 (3)	0.0719 (10)	
H5	0.3656	0.4419	0.4975	0.086*	
C3	0.3843 (3)	0.6372 (4)	0.5292 (3)	0.0856 (11)	
H4	0.4262	0.6279	0.5997	0.103*	
C4	0.3587 (3)	0.7608 (5)	0.4863 (4)	0.0922 (12)	
H3	0.3829	0.8367	0.5275	0.111*	
C5	0.2982 (3)	0.7738 (4)	0.3836 (4)	0.1035 (14)	
H2	0.2805	0.8589	0.3552	0.124*	
C6	0.2622 (3)	0.6628 (4)	0.3205 (3)	0.0831 (11)	
H1	0.2217	0.6736	0.2491	0.100*	
C7	0.3262 (2)	0.2968 (3)	0.2542 (3)	0.0544 (8)	
C8	0.3694 (2)	0.3417 (3)	0.1706 (3)	0.0674 (9)	
H10	0.3487	0.4182	0.1278	0.081*	
C9	0.4430 (2)	0.2742 (4)	0.1497 (3)	0.0769 (10)	
H9	0.4715	0.3062	0.0933	0.092*	
C10	0.4746 (2)	0.1605 (4)	0.2111 (4)	0.0815 (11)	
H8	0.5247	0.1159	0.1974	0.098*	
C11	0.4311 (3)	0.1143 (4)	0.2928 (3)	0.0859 (11)	
H7	0.4514	0.0367	0.3343	0.103*	
C12	0.3575 (2)	0.1808 (3)	0.3147 (3)	0.0728 (10)	
H6	0.3287	0.1477	0.3705	0.087*	
N1	0.02673 (18)	0.3481 (3)	0.3918 (2)	0.0648 (7)	
H1A	0.0801	0.3370	0.3811	0.078*	
N2	−0.00790 (19)	0.4687 (3)	0.2200 (2)	0.0800 (9)	
H13	0.0456	0.4558	0.2105	0.096*	
H14	−0.0458	0.5142	0.1695	0.096*	
C13	−0.0324 (2)	0.4193 (3)	0.3131 (3)	0.0638 (9)	
C14	−0.1181 (2)	0.4384 (4)	0.3339 (3)	0.0765 (10)	
H18	−0.1605	0.4888	0.2822	0.092*	
C15	−0.1392 (3)	0.3836 (4)	0.4294 (4)	0.0877 (12)	
H17	−0.1963	0.3965	0.4430	0.105*	
C16	−0.0765 (3)	0.3082 (4)	0.5071 (4)	0.0874 (12)	
H16	−0.0914	0.2690	0.5719	0.105*	
C17	0.0058 (3)	0.2930 (4)	0.4874 (3)	0.0789 (10)	
H15	0.0489	0.2442	0.5397	0.095*	
O3	0.16544 (17)	0.0543 (2)	0.44385 (19)	0.0746 (7)	
H3A	0.163 (2)	0.059 (3)	0.5239 (12)	0.090*	
H3B	0.181 (2)	0.1430 (16)	0.426 (3)	0.090*	

### Atomic displacement parameters (Å^2^)

**Table:**

	*U*^11^	*U*^22^	*U*^33^	*U*^12^	*U*^13^	*U*^23^
P1	0.0523 (5)	0.0634 (6)	0.0563 (5)	0.0041 (4)	0.0170 (4)	0.0055 (4)
O1	0.0624 (14)	0.0753 (15)	0.0752 (13)	−0.0004 (11)	0.0318 (12)	0.0181 (12)
O2	0.0537 (13)	0.0925 (17)	0.0555 (12)	0.0113 (12)	0.0084 (11)	0.0058 (11)
C1	0.0520 (19)	0.059 (2)	0.0570 (19)	0.0083 (16)	0.0193 (16)	0.0046 (16)
C2	0.083 (3)	0.065 (2)	0.065 (2)	0.003 (2)	0.010 (2)	0.0038 (19)
C3	0.090 (3)	0.088 (3)	0.075 (2)	0.000 (3)	0.011 (2)	−0.011 (2)
C4	0.083 (3)	0.077 (3)	0.119 (4)	−0.004 (2)	0.026 (3)	−0.026 (3)
C5	0.102 (4)	0.055 (3)	0.140 (4)	0.011 (2)	0.000 (3)	0.007 (3)
C6	0.087 (3)	0.060 (2)	0.095 (3)	0.011 (2)	0.005 (2)	0.012 (2)
C7	0.0497 (19)	0.056 (2)	0.0580 (18)	0.0007 (15)	0.0127 (16)	−0.0038 (16)
C8	0.060 (2)	0.075 (2)	0.071 (2)	0.0017 (18)	0.0221 (18)	−0.0022 (18)
C9	0.063 (2)	0.092 (3)	0.083 (2)	−0.011 (2)	0.031 (2)	−0.023 (2)
C10	0.062 (2)	0.092 (3)	0.092 (3)	0.017 (2)	0.020 (2)	−0.027 (2)
C11	0.084 (3)	0.080 (3)	0.093 (3)	0.024 (2)	0.020 (2)	−0.004 (2)
C12	0.070 (2)	0.076 (3)	0.075 (2)	0.011 (2)	0.0208 (19)	0.004 (2)
N1	0.0588 (18)	0.0722 (19)	0.0668 (17)	0.0040 (15)	0.0214 (15)	−0.0004 (15)
N2	0.0641 (18)	0.107 (2)	0.0696 (19)	0.0141 (17)	0.0156 (16)	0.0089 (17)
C13	0.061 (2)	0.072 (2)	0.059 (2)	−0.0053 (19)	0.0153 (19)	−0.0150 (18)
C14	0.056 (2)	0.099 (3)	0.076 (2)	0.005 (2)	0.0184 (19)	−0.012 (2)
C15	0.065 (3)	0.113 (3)	0.094 (3)	0.002 (2)	0.037 (2)	−0.018 (3)
C16	0.083 (3)	0.105 (3)	0.087 (3)	0.001 (2)	0.046 (3)	0.004 (2)
C17	0.084 (3)	0.082 (3)	0.075 (2)	0.000 (2)	0.027 (2)	0.005 (2)
O3	0.0917 (17)	0.0687 (15)	0.0640 (14)	−0.0134 (14)	0.0191 (13)	−0.0017 (12)

### Geometric parameters (Å, °)

**Table:**

P1—O2	1.498 (2)	C10—C11	1.367 (5)
P1—O1	1.508 (2)	C10—H8	0.9300
P1—C1	1.800 (3)	C11—C12	1.379 (5)
P1—C7	1.801 (3)	C11—H7	0.9300
C1—C6	1.376 (4)	C12—H6	0.9300
C1—C2	1.383 (4)	N1—C13	1.341 (4)
C2—C3	1.370 (5)	N1—C17	1.355 (4)
C2—H5	0.9300	N1—H1A	0.8600
C3—C4	1.356 (5)	N2—C13	1.329 (4)
C3—H4	0.9300	N2—H13	0.8601
C4—C5	1.351 (5)	N2—H14	0.8600
C4—H3	0.9300	C13—C14	1.398 (4)
C5—C6	1.376 (5)	C14—C15	1.353 (5)
C5—H2	0.9300	C14—H18	0.9300
C6—H1	0.9300	C15—C16	1.387 (5)
C7—C8	1.377 (4)	C15—H17	0.9300
C7—C12	1.386 (4)	C16—C17	1.337 (5)
C8—C9	1.379 (5)	C16—H16	0.9300
C8—H10	0.9300	C17—H15	0.9300
C9—C10	1.373 (5)	O3—H3A	0.954 (10)
C9—H9	0.9300	O3—H3B	0.953 (10)
			
O2—P1—O1	116.01 (13)	C8—C9—H9	119.5
O2—P1—C1	109.03 (14)	C11—C10—C9	118.7 (3)
O1—P1—C1	107.64 (13)	C11—C10—H8	120.7
O2—P1—C7	110.68 (13)	C9—C10—H8	120.7
O1—P1—C7	108.71 (14)	C10—C11—C12	121.1 (4)
C1—P1—C7	104.06 (14)	C10—C11—H7	119.5
C6—C1—C2	117.0 (3)	C12—C11—H7	119.5
C6—C1—P1	121.6 (3)	C11—C12—C7	120.4 (3)
C2—C1—P1	121.3 (2)	C11—C12—H6	119.8
C3—C2—C1	122.2 (3)	C7—C12—H6	119.8
C3—C2—H5	118.9	C13—N1—C17	122.7 (3)
C1—C2—H5	118.9	C13—N1—H1A	118.7
C4—C3—C2	119.3 (4)	C17—N1—H1A	118.7
C4—C3—H4	120.4	C13—N2—H13	120.3
C2—C3—H4	120.4	C13—N2—H14	119.7
C5—C4—C3	120.1 (4)	H13—N2—H14	120.0
C5—C4—H3	119.9	N2—C13—N1	119.7 (3)
C3—C4—H3	119.9	N2—C13—C14	122.9 (3)
C4—C5—C6	120.9 (4)	N1—C13—C14	117.5 (3)
C4—C5—H2	119.5	C15—C14—C13	120.0 (4)
C6—C5—H2	119.5	C15—C14—H18	120.0
C1—C6—C5	120.5 (4)	C13—C14—H18	120.0
C1—C6—H1	119.8	C14—C15—C16	120.5 (4)
C5—C6—H1	119.8	C14—C15—H17	119.7
C8—C7—C12	118.3 (3)	C16—C15—H17	119.7
C8—C7—P1	120.9 (2)	C17—C16—C15	118.9 (4)
C12—C7—P1	120.8 (2)	C17—C16—H16	120.5
C7—C8—C9	120.6 (3)	C15—C16—H16	120.5
C7—C8—H10	119.7	C16—C17—N1	120.4 (4)
C9—C8—H10	119.7	C16—C17—H15	119.8
C10—C9—C8	120.9 (4)	N1—C17—H15	119.8
C10—C9—H9	119.5	H3A—O3—H3B	104 (3)

### Hydrogen-bond geometry (Å, °)

**Table:**

*D*—H···*A*	*D*—H	H···*A*	*D*···*A*	*D*—H···*A*
N1—H1A···O1	0.86	1.80	2.655 (4)	175
O3—H3A···O2^i^	0.95 (2)	1.79 (2)	2.743 (3)	175 (3)
O3—H3B···O1	0.95 (2)	1.80 (2)	2.744 (3)	171 (3)
N2—H13···O2	0.86	2.02	2.881 (4)	176
N2—H14···O3^ii^	0.86	2.04	2.853 (4)	157

Symmetry codes: (i) *x*, −*y*+1/2, *z*+1/2; (ii) −*x*, *y*+1/2, −*z*+1/2.

## References

[bb1] Almeida, I. G. D. (1974). *J. Radioanal. Chem.* **22**, 21–28.

[bb2] Brandenburg, K. (2001). *DIAMOND* Crystal Impact GbR, Bonn, Germany.

[bb3] Espinet, P. & Soulantica, J. K. (1999). *Coord. Chem. Rev.* **193**, 499–556.

[bb4] Gentzkow, W. von, Huber, J. & Kapitza, H. (1996). US Patent No. 5 587 243.

[bb5] Huber, J., Kapitza, H. & Kleiner, H.-J. (1998). US Patent No. 5 811 188.

[bb6] Jeffrey, J. C. & Rauchfuss, T. B. (1979). *Inorg. Chem.* **18**, 2658–2666.

[bb7] Sheldrick, G. M. (2008). *Acta Cryst.* A**64**, 112–122. 10.1107/S0108767307043930 18156677

[bb8] Spek, A. L. (2009). *Acta Cryst.* D**65**, 148–155. 10.1107/S090744490804362X PMC2631630 19171970

[bb9] Stoe & Cie (2007). *X-AREA*, *X-RED* and *X-SHAPE* Stoe & Cie GmbH, Darmstadt, Germany.

